# An edge-based statistical analysis of long non-coding RNA expression profiles reveals a negative association between Parkinson’s disease and colon cancer

**DOI:** 10.1186/s12920-021-00882-6

**Published:** 2021-02-02

**Authors:** Suyan Tian, Mingyue Zhang, Zhiming Ma

**Affiliations:** 1 Division of Clinical Research, First Hospital of Jilin University, 1 Xinmin Street, Changchun, 130021 Jilin People’s Republic of China; 2 Department of Gastroenterology, First Hospital of Jilin University, 1 Xinmin Street, Changchun, 130021 Jilin People’s Republic of China; 3 Department of Gastrointestinal Nutrition and Hernia Surgery, Second Hospital of Jilin University, 218 Ziqiang Road, Changchun, 130041 Jilin People’s Republic of China

**Keywords:** Colon cancer, Parkinson’s disease, Long non-coding RNA (lncRNA), Weighted gene co-expression network (WGCNA), Edge, Association

## Abstract

**Background:**

Colon cancer (CC) is one of the most common malignant tumors, while Parkinson’s disease (PD) is the second most common neurodegenerative disorder. Recent accumulating evidence indicates that these two diseases are associated with each other. Also, from the perspective of long non-coding RNAs, some well-known genes such as H19 and PVT1 can link these two diseases together. Several studies have shown that patients with PD had a decreased risk of developing CC compared with patients without PD. However, controversies surround the relationship between PD and CC, and to date, no concordant conclusion has been drawn.

**Methods:**

In this study, we aimed to assess the association between these two diseases based on lncRNA-to-lncRNA interactions. Motivated by the weighted gene co-expression network analysis method, a customized procedure was proposed and used to identify differentially correlated edges (DCEs) in the respective interaction networks for PD and CC and explore how these two diseases are linked.

**Results:**

Of the two sets of DCEs for PD and CC, 16 pairs overlapped. Among them, 15 edges had opposite signs, with positive signs for CC indicating a gain of connectivity, whereas negative signs for PD indicating a loss of connectivity.

**Conclusions:**

By using the lncRNA expression profiles, and a customized procedure, an answer to the question about how PD and CC are associated is provided.

## Background

Long non-coding RNAs (lncRNAs) are non-coding RNAs with a length of more than 200 nucleotides, which are widely present in the genome [1]. They are post-transcriptional and epigenetic regulators having lower expression levels on average and are more tissue-specific when compared with protein-coding genes [1]. Recently, a variety of lncRNAs have been shown to possess diagnostic or/and prognostic values for complex diseases, including cancer and neurodegenerative diseases [1].

Colon cancer (CC), also known as colorectal cancer, is one of the most common malignant tumors. According to the Globocan 2018 data, CC is the fourth incident cancer as well as the second-highest leading cause of death in the world [2]. One recent study [3] showed that the number of differentially expressed lncRNAs between CC tissues and their adjacent normal tissues is not trivial. Therefore, same to other cancers, lncRNAs also play essential roles in the development and progression of CC.

**Fig. 1:**
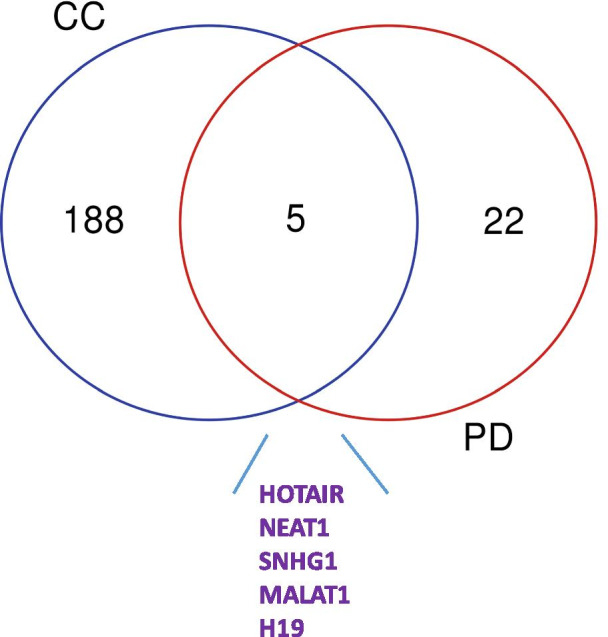
Venn-diagrams for experimentally validated lncRNAs that relate to Parkinson’s disease and colon cancer. *PD* Parkinson’s disease, *CC* colon cancer. Those lncRNAs were indicated to be experimentally validated by the lncRNADisease 2.0 database

Parkinson’s disease (PD) is the second most common neurodegenerative disorder, which mainly affects the elderly population aged over 65 [4]. Studies on the association between lncRNAs and PD are more recent than studies on the association between lncRNAs and cancer [5]. From the lncRNADisease 2.0 database [6], we downloaded the experimentally validated disease-to-lncRNA associations. Specifically, about 193 lncRNAs are associated with CC, and 27 lncRNAs are linked to PD. While the number of lncRNAs associated with CC is about seven times higher than that of PD, five lncRNAs (their gene symbols are given in Fig. 1) are shared by these two diseases, which motivated us to explore potential interplays between these two diseases from the perspective of lncRNA signatures.

Next, a literature search in the PubMed database with keywords “Parkinson’s disease” and “lncRNA” presented 104 papers while keywords “colon cancer” or “colorectal cancer” and “lncRNA” presented 501 papers. In addition to the five overlapped lncRNAs obtained in the lncRNADisease 2.0 search, more lncRNAs associated with both diseases have appeared, namely, *SNHG1*4 [7, 8], *UCA1* [9–11], and *P21* [12, 13]. The shared genes by these two diseases further support that it is reasonable to explore the links between PD and CC from the perspective of lncRNAs.

PD and CC share several common risk factors, among which aging is the predominant one, with substantially higher rates among people aged 60 and above. It is worth mentioning that more explicit and direct evidence of potential links between these two diseases comes from epidemiological studies [14–17]. With adequate and solid supports on that both PD and CC are related, there is one question—how are they correlated? In the above epidemiological studies, the association direction is not in agreement with one another. A majority of studies have stated that patients with PD had a decreased risk of developing CC, compared to those without PD; however, there are several exceptions. For example, a study from Taiwan [17] supported a positive association that an increased risk of developing CC in patients with PD compared to patients without PD.

Also, we notice that it is hard to state the direction of association based on the expression values of associated lncRNAs. Regarding the following lncRNAs—*H19, NEAT1, UCA1, HOTAIR,* and *SNHG1*, studies have shown that these are over-expressed in the tissues of CC than those of normal controls, while these are also up-regulated in PD patients. This led to the conclusion that PD and CC are positively correlated. Nevertheless, several in-depth studies discovered that aging acts as an intermitted factor that increases the risks of both PD and CC. These lncRNAs have been validated to play critical roles in aging. Therefore, an investigation on individual genes only cannot eliminate the confounding effects from those common risk factors such as aging, making the positive correlation between PD and CC superficial and non-confirmatory. Moreover, some studies have suggested that for specific individual lncRNAs, the direction of regulation might be opposite to each other in these two diseases. For example, it has been demonstrated that lncRNA *P21* is over-expressed in patients with PD [12, 18], while it is suppressed or unexpressed in patients with CC [13].

Unlike conventional gene-based methods, an edge/network method examines the interplaying among multiple genes, which are grouped together as an entire set to influence a biological process [19]. The edge methods focus on the second moment of expression values (in the network graph, it corresponds to an edge) that may convey more valuable information on the onset and progression of a complex disease, instead of the first moment (in the network, it corresponds to a node). Meanwhile, we think that such methods may also provide a more suitable alternative to identify the links between two distinct attributes—the potential relationship between PD and CC.

In this study, we addressed this issue by calculating lncRNA-to-lncRNA adjacency matrices to evaluate connections among genes. Then from the angle of edges instead of nodes, we explored the association direction between PD and CC. The differentially correlated edges (DCEs) were identified using a well-known bioinformatics tool—the weighted gene co-expression network analysis (WGCNA) [20] method—which is capable of building data-driven gene-to-gene interactions and is largely applied in the relevant fields, such as [21, 22]. Then by exploring if there are overlapped DCEs for these two diseases, their potential links and association directions are investigated.

## Methods

### Experimental data

For CC, the data of a microarray experiment in the Gene Expression Omnibus (GEO) repository (https://www.ncbi.nlm.nih.gov/geo/), which are available under the accession number GSE62932 [23] were considered. The chips of this experiment were hybridized on the Affymetrix HG-U133 Plus 2.0 platform. Since the number of controls in this experiment is small, we downloaded the expression profiles of controls from another microarray experiment, namely, GSE39582 [24], to mitigate the off-balance between the tumor samples and the normal samples. Notably, only control samples of this experiment were downloaded and then included in this study. The resulting integrated dataset included 64 patients with CC and 23 controls.

For PD, the experimental data of GSE7621 [25], whose chips were hybridized on the Affymetrix HG-U133 Plus 2.0 platform, were used to identify the differentially correlated edges. In this experiment, 16 diseased people and nine normal controls were included. The demographic characteristics of these experiments are summarized in Table 1.

**Table 1:** Characteristics of microarray experiments in this study

	Reference	Raw data	Platform	# of diseased people	Stages (I-IV)	Controls		Country
Parkinson’s disease								
GSE7621	Lesnick et al., PloS Genet, 2007	Yes	GPL570	16	–	9	USA	
Colon cancer								
GSE62932	Chen et al., PlosOne, 2016	Yes	GPL570	64	12/17/20/15	4	USA	
GSE39582	Marisa et al., Plos Med, 2013	Yes	GPL570	569*	NA	19	France	

*Only normal controls of this experiment were used

### Pre-processing procedures

Raw data (CEL files) of the above microarray datasets were downloaded from the GEO repository and pre-processed using the fRMA algorithm [26]. When there are multiple probe sets matched to the same gene, the probe set with the largest absolute log fold change (LFC) in comparison to the diseased group versus the control group was retained. For the CC cohort, since the samples of the control group came from two different datasets, the Combat algorithm [27] was implemented to adjust for potential batch effects.

By matching the gene symbols of lncRNAs in the GENCODE (https://www.gencodegenes.org/) database (version 32) to the genes annotated by the Affymetrix HG-U133 Plus 2.0 chips, the expression values of 2,299 probe-sets corresponding to 1,710 unique lncRNAs were input into the proposed edge method to identify differentially correlated edges in CC and PD cohorts, respectively.

### Statistical analysis

#### Identification of differentially correlated edges (DCEs)

Adopting the WGCNA method, we constructed the respective networks for the diseased groups and the control groups. Briefly, Pearson’s correlation coefficients (PCCs) or Spearman’s correlation coefficients (SCCs) were calculated for gene pairs. Next, a weighted network adjacency matrix was calculated by raising the absolute values of the correlation matrix to the power of *β* so that the resulting network is scale-free. By default, we observed that a value of 6 for *β* should make the under-construction networks satisfy this requirement.

In this study, we aimed to identify the differentially correlated edges in the diseased group versus the control group and the control group for CC and PD cohorts. First, for the CC cohort, the differentially correlated edges (DCEs) were identified by taking the absolute value of the difference in adjacency matrices of these two groups. If this value of the edge *ij* (which connects lncRNA *i* and lncRNA *j*) is greater than a pre-determined threshold, then the specific edge is regarded as a DCE between the diseased group and the control group. Otherwise, it is not a DCE. Likewise, the DCEs for PD cohort were identified using the same procedure. Finally, to link these two distinct diseases, the overlaps of these two DCE sets were explored. The sign of the difference between adjacency matrices of the disease group and the control group serves as an indicator, with a plus sign for gaining connections and a minus sign for losing connections between the corresponding edges. Of note, the SCCs were calculated during the construction of adjacency matrices in this study given the sample sizes of microarray experiments under consideration were not very large; thus, the normality assumption might not be valid.

### Identification of hub genes

The Cytoscape plugin cytoHubba was utilized to identify the hub genes that may play crucial roles in the resulting networks. Here, the top 50 genes ranked by their connectivity degree were regarded as the hub genes. The resulting gene-to-gene interaction networks were visualized with the aid of Cytoscape software.

### Enrichment analysis for target mRNAs

For the lncRNAs involved in overlapped DCEs between PD and CC, their target mRNAs were retrieved from the lncRNADisease 2.0 database [6]. Then, the String software [28] was used to perform pathway enrichment analysis and to retrieve interaction information of the target mRNAs by these identified lncRNAs. Also, the target mRNAs by the hub genes of the lncRNA-to-lncRNA interaction networks were retrieved from the lncRNADisease 2.0 database [6], and the String software was used to construct the gene-to-gene interaction networks and perform pathway enrichment analysis.

### Biological relevance

The Genecards database [29] was searched to explore the biological relevance of the lncRNAs involved in the identified DEGs with CC and PD. Furthermore, a PubMed search was carried out to retrieve very recent literature on the relevance of the identified genes to either CC or PD.

### Statistical language and packages

All statistical analysis was performed in the R language version 3.5 (www.r-project.org).

## Results

### DCEs for CC

Using the proposed procedure, the differentially correlated edges were first identified for the patients with CC. The cutoff value is set as 0.3, corresponding roughly a high correlation coefficient (its absolute value is at least above 0.8) in one group versus a mild or moderate correlation in the other group. Most signs of identified 284 DCEs (263/284) are positive, indicating a gain of connectivity for the patients with CC.

### DCEs for PD

Using the same cutoff, there were 19,785 DCEs for the PD application. Among this large number of DCEs, interestingly, most of their signs (19,342/19,785: 97.76%) were negative. For PD, many existing edges among genes have disappeared during the progression of the disease.

Of these two sets of DCEs, 16 pairs were overlapped and listed in Table 2. Among them, only one had the same positive sign for both CC and PD, while the remaining 15 overlaps had opposite signs. This partially justified that these two diseases may be negatively related.

**Table 2:** Overlapped differentially-correlated edges by colon cancer and Parkinson’s disease

Node 1	Node 2	PD	CC
FLJ40288	LOC101928144	1	1
LINC01365	LINC00868	− 1	1
LINC01210	LOC101929441	− 1	1
MIR4300HG	LINC00523	− 1	1
LINC00642	LOC101928443	− 1	1
LINC00642	MIR4313	− 1	1
LOC105375875	PRRX2-AS1	− 1	1
LOC101929657	SLFNL1-AS1	− 1	1
LOC101928443	MIR4313	− 1	1
LOC101928443	LOC283194	− 1	1
OGFRP1	LOC100996694	− 1	1
NAV2-AS5	LOC101929441	− 1	1
LOC101928476	LOC101929441	− 1	1
LOC283194	GABRG3-AS1	− 1	1
DTX2P1-UPK3BP1-PMS2P11	MORC2-AS1	− 1	1
MIR100HG	LOC339803	− 1	1

*CC* colon cancer, *PD* Parkinson’s disease. − 1 indicated a lose of connectivity while 1 means a gain of connectivity

### Biological relevance

Among the 25 lncRNAs involved in the overlapped DCEs for PD and CC, their biological relevance with CC and PD was investigated by searching the GeneCards database [29] and the lncRNADisease 2.0 database [6]. It is worth mentioning that most of these lncRNAs are largely unexplored, even some of them lack annotations by the corresponding databases. This was expected given that the edge method is capable of selecting insignificant (with subtle expression change in groups) but truly relevant “dark matter”.

GeneCards mining did not present many positive results. Specifically, only one lncRNA was indicated to be directly associated with CC, while five were indirectly associated, but the confidence scores for these associations were all extremely small. For PD, five lncRNAs were identified to be indirectly associated, and the confidence scores were small as well. Of these, either directly or indirectly related lncRNAs, three lncRNAs—*OGFRP1*, *LOC339803*, and *MIR100HG* were shared.

On the other hand, the lncRNADisease 2.0 mining indicated that nine lncRNAs—*FLI40288*, *LINC01365, LINC01210*, *MIR4300HG*, *LINC00642*, *PRRX2-AS1*, *OGFRP*, *MORC2-AS1,* and *MIR100HG* were correlated with CC and Alzheimer’s disease (but not PD) using computational biology methods. In summary, no solid evidence of biological relevance could be obtained by searching only on these identified lncRNAs.

Next, we used target mRNAs by these lncRNAs for possible biological relevance. As mentioned above, several of the identified lncRNAs have not been annotated yet; only 22 target mRNAs were found because of little exploration of these lncRNAs. For these 22 mRNAs, four were directly related to CC, and the remaining were indirectly related. Meanwhile, nine were directly associated, and the remaining ones were indirectly associated with PD according to the GeneCards database [29]. In this study, we only focused on the direct relevant ones (Table 3) and observed that *PTGES*, targeted by *PRRX2-AS1* was the only one common gene in these two subsets. On the other hand, a PubMed search for the recent studies was carried out, which added *CALM1* [30] and *GABRG3* [31] to this list (the list of being associated with both CC and PD with experimental validations).

**Table 3:** Directly associated mRNAs targeted by the lncRNAs involved in the overlapped differentially correlated edges by these two diseases

Symbol	Description	Score
Parkinson’s disease		
TOR1A	Torsin Family 1 Member A	32.27
GABRG3	Gamma-Aminobutyric Acid Type A Receptor Subunit Gamma3	5.45
CALM1	Calmodulin 1	4.37
CTPS1	CTP Synthase 1	4.17
** PTGES**	**Prostaglandin E Synthase**	**3.85**
BEGAIN	Brain Enriched Guanylate Kinase Associated	1.91
NTMT1	N-Terminal Xaa-Pro-Lys N-Methyltransferase 1	1.91
ASB6	Ankyrin Repeat And SOCS Box Containing 6	1.91
SLFNL1	Schlafen Like 1	1.91
Colon cancer		
MORC2	MORC Family CW-Type Zinc Finger 2	6.87
** PTGES**	**Prostaglandin E Synthase**	**2.32**
NAV2	Neuron Navigator 2	2.21
DLK1	Delta Like Non-Canonical Notch Ligand 1	0.25

Score: the confidence score given by the GeneCards database to indicate the strength of the underlying relevance. Here, only PTGES (highlighted in bold) is common by these two subsets

### Pathway enrichment analysis

The pathway enrichment analysis was performed using String [28] software. KEGG pathways [32] and GO terms [33] indicated that those target mRNAs only enriched one GO biological process (BP) term — *GO0071763 nuclear membrane organization*. Moreover, the String [26] software demonstrated that two gene pairs interacted with each other – *BEGAID* & *DLK1* and *TOR1A* & *TOR1B*.

### Hub genes for the differentially correlated networks

**Fig. 2:**
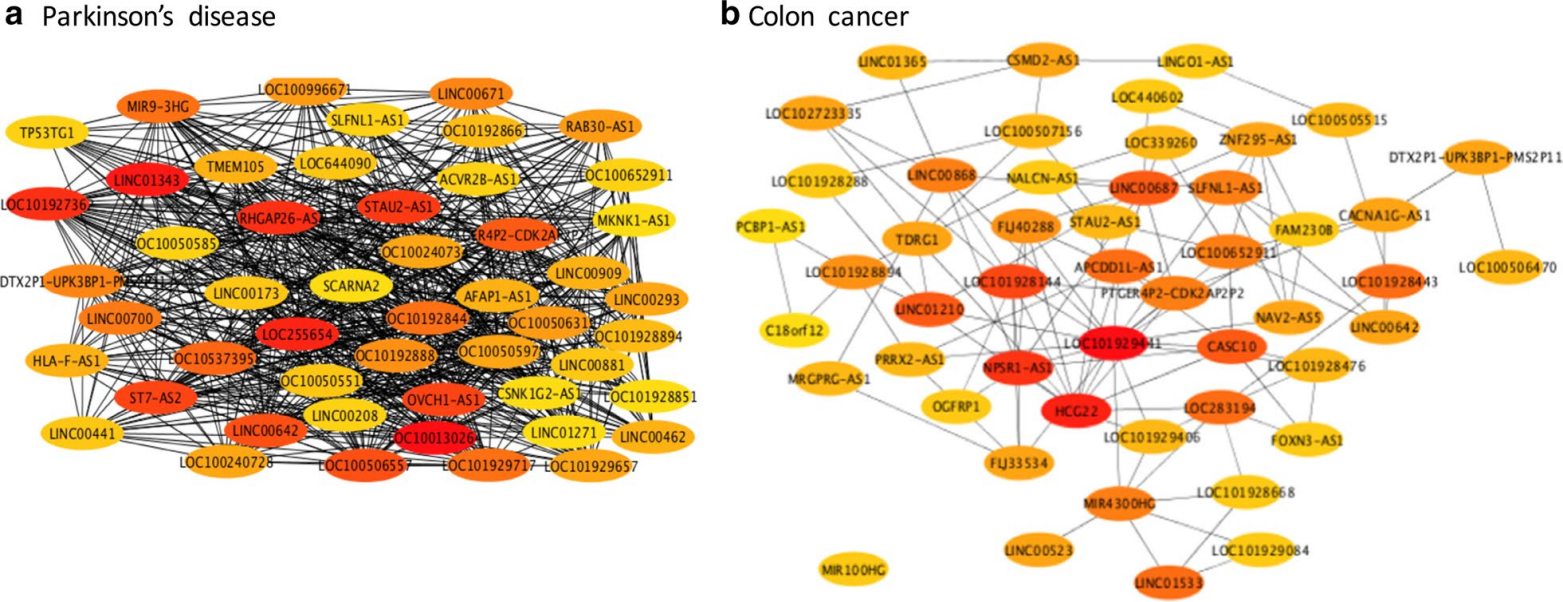
Top 50 hub genes of the resulting lncRNA-to-lncRNA interaction networks. **a** For Parkinson’s disease. **b** For colon Cancer. From these two sub-networks, we observe that the network for PD is very dense. In contrast, the network for CC is much sparse. *PD* Parkinson’s disease, *CC* colon cancer

Using the Cytoscape plugin cytoHubba, the respective 50 hub genes for the resulting differentially correlated networks of PD and CC were obtained (Fig. 2). Of these two sets of hub genes, nine lncRNAs—*LOC101928443, PTGER4P2-CDK2AP2P2, LOC100652911, STAU2-AS1, SLFNL1-AS1, LOC101928894, LINC00642, DTX2P1-UPK3BP1-PMS2P11,* and *LOC100505515* were overlapped. Unfortunately, these nine lncRNAs are largely unexplored.

**Fig. 3:**
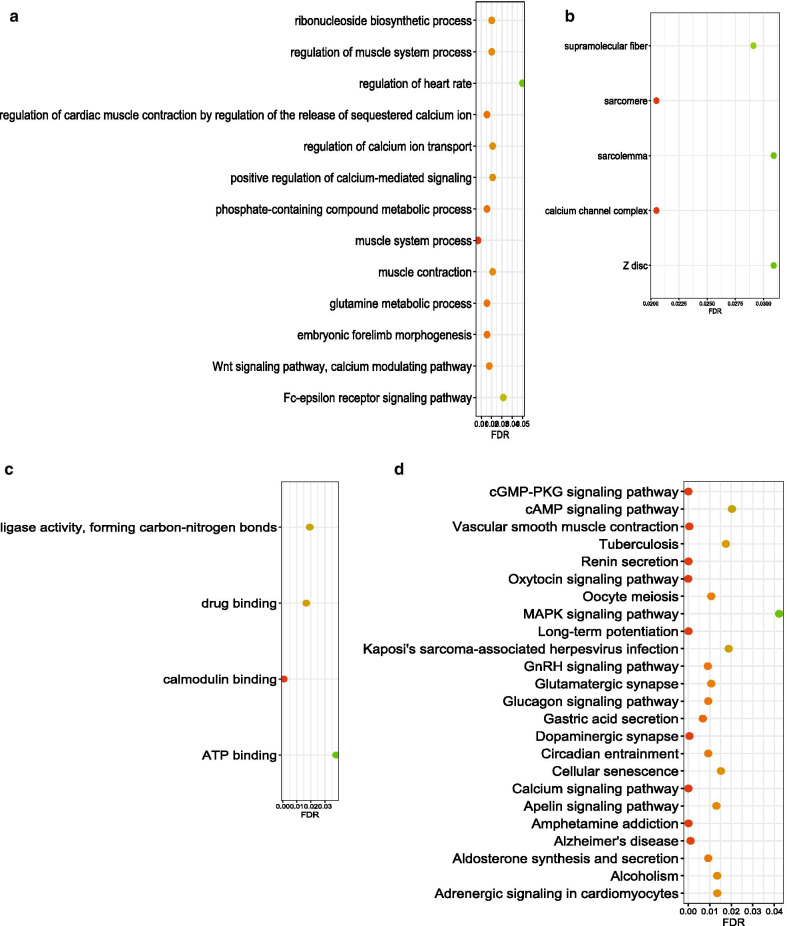
Functional analysis of the target mRNAs by overlapped hub lncRNAs between Parkinson’s disease and colon cancer. **a** For enriched GO cellular component terms. **b** For enriched GO molecular function terms. **c** For enriched GO biological process terms. **d** For enriched KEGG pathways. *GO* gene ontology, *KEGG* kyoto encyclopedia of genes and genomes

The target mRNAs by the overlapped hub genes were retrieved from the lncRNADisease 2.0 database. Of the target mRNAs, only one gene—*CALM1* has been reported to correlate with both CC [28] and PD [34, 35]. Then, the enriched pathways by these target mRNAs were identified using String software. Figure 3 presents the enriched KEGG pathways and GO terms, along with their corresponding FDRs. Of these pathways, many have been found to be associated with both PD and CC, such as *MAPK signaling pathway* [36, 37], *ATP binding pathway* [38, 39], and *cAMP signaling pathway* [40–42]. Therefore, the investigation of the hub genes harnessed more evidence to support the link between PD and CC.

## Discussion

Although solid research evidence guarantees a link between PD and CC, the question about how these two diseases are associated contradict one another in the literature. Unlike conventional gene-based bioinformatics tools, the network-based methods focus on the interplay among genes [21, 22], thus throwing light on this question from a different point of view.

In this study, we utilized a network-based procedure in which the essential component is WGCNA to identify DCEs for PD and CC. The results indicated that for CC, most of the identified gene pairs had plus signs, meaning a gain of connection between the corresponding edges, while PD tended to lose connections. This shows that PD and CC are negatively correlated, which is consistent with the results of most existing studies, such as [43, 44].

Furthermore, even though the resulting lncRNA list that was overlapped by PD and CC analyses included many unexplored or underexplored genes and thus lacked meaningful biological interpretation, the enriched pathways by the target mRNAs of overlapped hub lncRNAs involved many signaling pathways that are correlated to both PD and CC. As a result, experimental validation and further exploration of these hub genes are highly desirable.

One question that interests the researchers the most is about the causality relationship between PD and CC. In the literature, it is found that the patients with CC have a lower risk to develop PD while for PD patients, the probability of developing colon cancer decreases as well, implying they may be preventive factors for each other. Therefore, it is anticipated that the arrow between these two diseases might be bidirectional. Based on cross-sectional gene expression profiles, however, the justification of this conjecture is very difficult or even impossible. In addition, the patients should be followed up for a long period of time; a suitable statistical method is highly desirable. Such methods should be able to infer the regulation directions among genes and diseases, which will be the focus of our future research.

It is worth pointing out that the proposed procedure is based on the WGCNA method, which may be too simple to provide a perfect inference in some cases. In the future, we will try to adopt more advanced methods such as the deep learning method proposed by Liu et al. [45] to explore the potential link between these two diseases.

## Conclusions

In conclusion, we explored the lncRNA expression profiles using a customized bioinformatics method that is based on the WGCNA method and found a sensible answer to the question of how PD and CC are linked.

## Data Availability

Raw data of three microarray datasets—GSE7621, GSE62932 and GSE39582 were downloaded from the GEO (https://www.ncbi.nlm.nih.gov/geo/) database, all data are open and publicly available.

## Abbreviations

WGCNA: Weighted gene co-expression network analysis
CC: Colon cancer
PD: Parkinson’s disease
lncRNA: Long non-coding RNA
SCC: Spearman’s correlation coefficient
DCE: Differentially correlated edge
